# Non-Contrast Computed Tomography Scan Based Parameters of Ureteric Stones Affecting the Outcome of Extracorporeal Shock Wave Lithotripsy

**DOI:** 10.7759/cureus.1227

**Published:** 2017-05-05

**Authors:** Muhammad Waqas, Mohammad Ayaz Khan, Muhammad Waqas Iqbal, Mian Khalid Akbar, Imad-ud-din Saqib, Saeed Akhter

**Affiliations:** 1 Department of Urology, Shifa International Hospital, Islamabad, Pakistan; 2 Department of Plastic Surgery, Shifa International Hospital, Islamabad, Pakistan

**Keywords:** hounsfield unit, skin to stone distance, hounsfield density, extra corporeal shock wave lithotripsy, ureteric stone

## Abstract

**Objective:**

To compare the non-contrast computed tomography (NCCT) scan-based parameters of ureteric stones affecting the outcome of extracorporeal shock wave lithotripsy (ESWL).

**Materials and methods:**

We retrospectively evaluated the pre-procedure NCCT of 74 patients who had ESWL for solitary ureteric calculi of 5-20 mm in diameter. We assessed the age, sex, basal metabolic index (BMI), laterality, location, presence of double 'J' (DJ) stent, skin to stone distance (SSD), stone maximum diameter, Hounsfield unit (HU), Hounsfield density (HD), area, and volume. All those who had no stone on follow-up imaging within 30 days were declared successful while those who had residual stone were declared failures.

**Results:**

The overall success rate was 78% (58/74). Sixty (81.1%) patients were male. The success of ESWL was correlated with lower SSD, Hounsfield units (HU) and Hounsfield density (HD). However, in multivariate analysis, SSD, Hounsfield unit, and stone area showed correlation with success of procedure but Hounsfield density failed to show correlation. The success rate in patients with stone HU <500, 500-1000 and >1000 were 93.9%, 69%, and 58.3%, respectively. Patients with lower BMI (<30 kg/m^2^) and HD (<76 HU/mm) were more prone towards success of the procedure than those with higher BMI (>30 kg/m^2^) and higher HD (>76 HU/mm).

**Conclusion:**

BMI, SSD, stone Hounsfield units and Hounsfield unit density were strong predictors of outcome of ESWL for ureteric stone.

## Introduction

Urinary stone, also called nephrolithiasis, is one of the most common pathologies in medicine that is expected to involve five percent of women and 12% of men in their life time [1]. Since Pakistan is located on the stone belt region, there is a high incidence of urolithiasis [2-3]. Stone disease accounts for more than half of the patients in the outpatient department and more than one third of all urological admissions in a tertiary health center in Pakistan [3]. It is usually found in the fourth and fifth decade of life. Its location in the urinary tract varies. In developed countries, 97% of the stones are found in the kidney and the ureter. Among them, 59% are located in the ureter and 75% in the iliac and the pelvic part of the ureter. Treatment of urolithiasis includes conservative management, surgical management, and ESWL depending upon the evaluation by a professional medical team [1]. ESWL, after its introduction in 1980, has revolutionized the treatment of urinary stones. It is a noninvasive, outpatient procedure without the need for anesthesia [4]. It is considered as the first-line treatment of urinary tract stones with a success rate of 80-90% [1].

Currently, imaging is important not only for initial diagnosis but also for treatment planning and monitoring of patients with urolithiasis. After its introduction for stones in the 1990s, NCCT has become the gold standard modality for diagnosis and subsequent evaluations of patients with renal and ureteric stones. Its advantages are its high sensitivity and specificity (> 95% and > 96%, respectively), easy availability, faster speed of acquisition and avoidance of intravenous contrast [5]. NCCT not only provides information regarding urinary tract abnormalities but also aids in determining the stone location, size, shape, density, and skin to surface distance [6]. Multiple studies have investigated various parameters and their relationship to success rate of ESWL but with non-homogenous results. The studies in which these parameters were used only for ureteric stones are very few. In the our study, we focused on different NCCT scan-based parameters and their effects on the success of ESWL treatment for ureteric stones.

## Materials and methods

We retrospectively reviewed the records of 245 patients who underwent ESWL for ureteric stones between January 2014 to December 2016. The indication for ESWL includes stones > 5 mm which are not causing urinary obstruction. Patients were excluded if they had any of the following: 1) maximum stone length greater than 20 mm, 2) absolute contraindication to ESWL such as pregnancy or coagulopathy, 3) previous history of ureteral surgery, 4) structural urinary tract abnormality, 5) solitary kidney, 6) lost to follow-up, 7) developed urinary obstruction requiring surgical intervention, and 8) underwent a surgical procedure such as percutaneous nephrolithotomy (PCNL) or endoscopic treatment before ESWL. Only 74 patients were able to qualify for the study.

Before ESWL, each patient was routinely assessed. A medical history, physical exam, urinalysis, urine culture, serum chemistry profile and coagulation profile were performed in each patient. NCCT was done before ESWL in each case. ESWL was performed by a urologist. The number of shock waves and energy settings were decided by the urologist.

A maximum of 4000 shock waves were administered with a maximum power of 18 kV. The position of the patient was decided on the basis of stone location. Those who had upper ureteric stones were treated in the supine position while those with lower ureteric stones were treated in the prone position. The stones were fragmented under fluoroscopic and/or ultrasound guidance. During the procedure, the patients were monitored for pulse, blood pressure, and oxygen saturation. The pain was managed with intravenous nalbuphine. After the procedure, the patients were prescribed diclofenac sodium and tamsulosin. The patients were reviewed one week after the session with an X-ray KUB and/or ultrasonography (USG). NCCT was done in those patients who had radiolucent stones or the stones were not visible on USG. The procedure was repeated if there was no fragmentation or inadequate fragmentation (stone fragment > 4 mm).

ESWL was considered successful if there was no stone on imaging studies at the one-week follow-up visit. The patients were then followed-up on the 14th, 21st and 30th day. Subsequent sessions of ESWL were decided based on the clearance of stone. Stones which showed no or poor fragmentation after 30 days were considered as failure of ESWL. Success was defined in the case when no stones were detected on post procedure imaging.

A multidetector CT scan Aquilion One, 3.0 mm/ 120Kv/ 200mAs/ Merge Healthcare 2006 & 2010 (Toshiba Medical Systems, Japan) was used. All radiological parameters were measured by a post graduate resident. Maximum stone diameter was measured in the radiograph with the largest stone diameter. Hounsfield units (HU) were measured by creating three regions of interest in three different views of the stones on CT scan showing the stone in the largest dimension. The average of the three regions of interest was considered a representation of the HU for that stone. Care was taken to exclude soft tissue in the measurements.

Skin to surface distance (SSD) was considered an average of the measurements of the distance between skin and stone at 00, 450, and 900 in transverse section of NCCT. The area of the stone was measured by tracing the contour of the inner edge of the stone (not including the surrounding soft tissue) on the slice with maximum stone cross sectional area. The stone volume was calculated by SV = l. w. d. π. 0.167 (π =3.14159) [7].

All statistical analyses were performed by using the Student’s t-test, analysis of variance (ANOVA), and Pearson’s chi-square test. SPSS ver. 16.0 (SPSS Inc., Chicago, IL, USA) was used, and p-values less than 0.05 were considered statistically significant.

## Results

Among 74 patients, 58 (78%) had successful clearance of stone. Among them, 60 (81.1%) were male. Twenty-four patients had double 'J' (DJ) stents in situ when ESWL was done. The mean age and BMI were 42.6 ± 12.21 years and 28.07 ± 4.86 kg/m2. Most of the stones (79.7%) were located in the upper ureter. The groups of patients whose procedures were successful and those whose procedures were failures were well matched for age, sex, stone size, and stone location. There was no significant difference in mean BMI, number of shock waves delivered, mean number of sessions, mean maximum length of stone, area and volume of stone between the two groups (Table 1).

SSD between successful and failure groups were 119.30 ± 17.71 mm and 130.75 ± 13.11 mm (p 0.008). The mean HU and Hounsfield unit density (HD) of stones were significantly lower in the successful group than in the failure group (HU = 546.10 ± 221.75 HU vs 915.55 ± 261.20 HU p 0.000 and HD = 66.9105 ± 35.64 HU/mm vs 90.16 ± 32.06 HU/mm, p 0.019) (Table 1).

**Table 1:** Overall characteristics of patients SD Standard deviation, IQR Interquartile range, ¶ Chi square test, † Independent T test, ‡ Mann-Whitney U test.

Variables	Overall	Successful	Failed	P value
Patients (n)	74	58 (78.4%)	16 (21.6%)	
Age (years) Mean±SD	42.6±12.21	42.1034±11.86	44.4375±13.66	0.541^†^
Gender Male​ Female	60 (81.1%) 14 (18.9%)	46 (76.7%) 12 (85.7%)	14 (23.3%) 2 (14.3%)	0.459 ^¶^
BMI (kg/m^2^)	28.07±4.86	27.63±4.71	29.69±5.21	0.166^†^
DJ stent Yes No	24 (32.4%) 50 (67.6%)	19 (79.2%) 39 (78%)	5 (20.8%) 11 (22%)	0.909 ^¶^
Previous Lithotripsy Yes No	10 (13.5%) 64 (86.5%)	9 (90%) 49 (76.6%)	1 (10%) 15 (23.4%)	0.337 ^¶^
Opacity Lucent Faint Opaque	9 (12.2%) 14 (18.9%) 51 (68.9%)	7 (77.8%) 14 (100%) 37 (72.5%)	2 (22.2%) 0 (-) 14 (27.5%)	0.087 ^¶^
Laterality Left Right	39 (52.7%) 35 (47.3%)	29(74.4%) 29 (82.9%)	10 (25.6%) 6 (17.1%)	0.375 ^¶^
Location Upper ureter Mid ureter Lower ureter	59 (79.7%) 7 (9.5%) 8 (10.8%)	45 (76.3%) 7 (100%) 6 (75%)	14 (23.7%) 0 (-) 2 (25%)	0.343 ^¶^
Number of sessions (mean) Mean±SD	1.51±0.84	1.50±0.88	1.56±.72	0.774^†^
Mean energy level (kv) Mean±SD	18.39±1.08	18.40±1.09	18.37±1.09	0.945^†^
Number of shock waves Median (IQR) Mean±SD	4000(4025) 5894.59±5153.82	4000(4200) 6029.31±5621.13	4000(4000) 5406±2978.92	0.816^‡^
Skin to stone distance Median (IQR) Mean± SD	122.10(20.84) 121.78±17.40	119.65(20.14) 119.30±17.71	127.66(21.62) 130.75±13.11	0.014^‡^
Mean Hounsfield density (HU/mm) Median (IQR) Mean± SD	68.06(49.16) 70.87±35.12	59.23(49.36) 65.54±34.38	82.32(33.09) 90.16±32.06	0.007^‡^
Mean Hounsfield units Median (IQR) Mean± SD	545.0(377.59) 625.97±275.43	474.50(234.05) 546.10±221.75	928.67(402.00) 915.55±261.20	<0.001^‡^
Maximum diameter of stone (mm) Median (IQR) Mean± SD	9.28(5.41) 9.64±3.42	9.22(5.3) 9.40±3.38	9.66(6.02) 10.50±3.50	0.217^‡^
Stone Volume (mm^3^) Median (IQR) Mean± SD	197.42(242.14) 238.81±222.34	155.51(245.49) 227.64±225.90	206.67(195.88) 279.29±210.81	0.152 ^‡^
Stone Area (mm^2^) Median (IQR) Mean± SD	34.67(38.27) 39.41±27.30	28.75(42.88) 39.53±29.62	37.89(25.13) 38.95±17.10	0.462 ^‡^

The success rate in patients with BMI < 30 kg/m2 was higher (88.7%) than in patients with BMI > 30 kg/m2 (52.4%) (p 0.001). Similarly, there was a higher success rate (81.4%) in patients with stone size < 10 mm compared to those with stone size > 10 mm (74.2%) though without any significant correlation (p 0.458).

We divided the patients into three groups i.e. HU < 500, 500 to 1000, and > 1000. The success rate decreased with an increase in Hounsfield unit of stone, i.e. 31 (93.9%), 20 (69%), 07 (58.3%), respectively, (p 0.011). We found that 37/74 (88.1%) patients with Hounsfield density < 76 HU/mm were successful in comparison with 21/74 (65.6%) patients with HD > 76 HU/mm (p 0.02) (Table 2).

**Table 2:** Effect of different group variables on the success of ESWL ¶ Chi square test

Variables	Successful group	Failure Group	P Value
BMI (kg/m^2^) <30 >30	47(88.7%) 11 (52.4%)	06 (11.3%) 10 (47.6%)	0.001^¶^
Maximum diameter of stone (mm) <10 mm >10mm	35 (81.4%) 23 (74.2%)	08(18.6%) 08 (25.8%)	0.458^¶^
Hounsfield units (HU) <500 500- 1000 >1000	31 (93.9%) 20 (69%) 07 (58.3%)	02 (6.1%) 09 (31%) 05 (41.7%)	0.011^¶^
Hounsfield density (HU/mm) <76 >76	37 (88.1%) 21 (65.6%)	05 (11.9%) 11 (34.4%)	0.020^¶^

Table 3 details the prediction of stone-free rate on the basis of the results of this study using multivariate analysis.

**Table 3:** Multivariate analysis of variables predicting the stone-free rate by logistic regression analysis

Variables	p value	Odds ratio	95.0% Confidence Interval	
			Lower	Upper
SSD	0.047	1.080	1.001	1.165
Hounsfield units	0.022	1.011	1.002	1.020
Hounsfield density	0.431	0.969	0.894	1.049
Maximum diameter of stone	0.912	1.040	0.520	2.080
Stone volume	0.809	0.999	0.993	1.005
Stone area	0.042	10.913	0.837	0.997

## Discussion

Since its inception, ESWL has become the treatment of choice for both renal and upper ureteric stones. However, nowadays, with newer lithotripters and better imaging modalities, ESWL is also considered suitable for middle and lower ureteric stones as for renal and upper ureteric stones [8].

Factors which affect the success of ESWL can be categorized as stone factors (including stone size, location, composition, degree of obstruction, skin to stone distance, stone attenuation value), clinical factors (solitary kidney, abnormal ureteral anatomy and comorbidities such as concomitant infection) and technical factors (type of lithotripter, source of energy) [9].

Non-contrast CT scan has become the imaging investigation of choice not only for the detection of urinary stones but it also plays a role in deciding the modality of treatment and predicts the outcome of that treatment [10-11]. We evaluated the effect of NCCT determining parameters on the success of ESWL for ureteral stones. Overall reported stone clearance with ESWL for ureteric stones ranges from 73 to 86% [12] which matched the 78% stone clearance in our study.

Abdelghany M, et al. reported obesity (BMI > 30 kg/m^2^) as a negative predictor for ESWL for lower ureteric stones. They described chances of tenfold increased failure rate in patients with BMI > 30 kg/m^2^ than those with BMI < 30 kg/m^2^ after two sessions of ESWL [12]. In another study, stone-free rate for normal weight and morbidly obese patients for upper ureteric stones were 82% and 67%, respectively [13]. Müllhaupt, et al. suggested the cut-off values of BMI and weight as 25.9 kg/m^2^ and 82.5 kg for ESWL outcome prediction [14]. In our study however, higher BMI was associated with an increased chance of failure of ESWL, but the difference was not statistically significant. On the other hand, when we divide the patients into two groups, one with BMI more than 30 kg/m^2^ and the other with BMI less than 30 kg/m^2^, the stone clearance was 88.7% in patients with BMI < 30 kg/m^2^ while it was only 52.4% in those with BMI > 30 kg/m^2^ (p 0.001).

Many investigators have suggested SSD as an important predictor of ESWL outcome [11, 14-15]. Park BH, et al. described significant influence of SSD for ESWL in renal stones [6]. Müllhaupt, et al. studied the effect of SSD on ureteral stone fragmentation and described its significance. The SSD 90^0^ with a cut off value of 11.9 cm was a stronger predictor of stone fragmentation than SSD 0^0^ and 45^0^ [14]. Other studies failed to show a similar effect of SSD on stone clearance with ESWL [10-11]. Our study demonstrated that higher SSD is a bad prognostic factor for clearance of ureteric stones. SSD was significantly lower in the successful group (119.65 mm) in comparison with the failure group (127.66 mm) (p 0.014). This might be due to the fact that shock waves have to travel larger distance in patients with larger BMI and SSD, which results in loss in energy of waves and decreased ability to disintegrate the stones.

Larger stones have been reported to be associated with higher risk for failure of urinary stone treatment. Ureteral stones larger than 10 mm are more prone to failure of ESWL therapy. Choi JW, et al. described the success rate of 90.2% for stone size < 10 mm and 68.6% for stone size > 10 mm [15]. Another study reported 86.8% and 70.8% success rate for lower ureteric stones of < 10 mm and > 10 mm, respectively [12]. The success of the procedure is not only related with the stone size, rather there is also an association with the number of shockwaves and the number of sessions [9]. In our study, the success rate was 81.4% for stones < 10 mm and 74.2% for stones > 10 mm without any statistical difference between the rates. Similarly, maximum stone diameter was higher in the failure group than in the successful group (9.22 vs 9.66 mm) having no statistical significance.

Stone attenuation is based on the principle of measuring the amount of X-rays passed through or absorbed by the tissue. It is an important parameter not only in predicting the composition of urinary stones but also in determining the preferred mode of treatment [16]. Stone attenuation value has been correlated with the fragility of stones. Stone density measurement is easy, reliable, objective, and reproducible. Abdelaziz, et al. in his study included both renal and ureteric stones and described 100% and 32% success rates associated with HU < 500 and > 800, respectively. They described the stone HU > 800 as a bad prognostic factor for ESWL success [10].

Ouzaid, et al. in his prospective analysis of 50 patients described the HU threshold of 970 for predicting the outcome of ESWL. They showed a success rate of 96% and 38% for stones with HU < 970 HU and > 970 HU, respectively [17]. Yazici O, et al. also demonstrated a significant effect of Hounsfield units on stone-free rate for proximal ureteric stone values (mean: 702 HU and 930 HU, p < 0.0001) with an inability to demonstrate similar relationship for distal ureteric stones [18]. Sultan M, et al. in their prospective analysis of 100 patients with renal stones showed a success rate of 100% for HU < 500, 96% for HU 500-1000, and 0% for HU > 1000. They also analysed the relationship between increased Hounsfield units with increasing requirement of number of shockwaves [19].

In another study, the authors concluded that patients with stone attenuation value > 956.5 HU should not be considered for EWSL particularly in those with BMI > 30 [20]. In some studies, however, no significant correlation was found between Hounsfield unit value and ESWL outcome [6, 11]. In our study, Hounsfield units of stone was a strong predictor of success of the procedure. Median Hounsfield units in the successful group was 928.67 HU as compared to 474.50 HU in the failure group (p < 0.001). The success rate was 93.9% for stones with HU < 500 while it decreased to 58.3% for HU > 1000 (p 0.011). When a comparison was made between the various NCCT scan parameters, statistical significance was noted once again.

In our results, we found that stone area was not a predictor of ESWL outcome. However, multivariate analysis showed correlation of stone area with the success of procedure. Tanaka, et al. in their analysis of upper urinary stones found that both the stone attenuation value and the stone area are good predictors of ESWL outcome. They noted that patients having stone attenuation value of 780 HU or less and a stone cross sectional area of 0.4 cm^2^ or less have 1.16 times more chances of ESWL success [21].

Composition of stone is considered the most important factor in determining the appropriate treatment option for urinary stone. Motley G concluded that HD is a better predictor of composition of stones than HU. It was observed that all stones with HD >76 HU/mm were calcium stones [22]. Yazici O, et al. described the mean HD in the stone-free group and residual stone group as 81 ± 31 HU/mm and 89 ± 25 HU/mm, respectively, for distal ureteric stones, which showed no statistical significance [18].

In our study, median HD in the successful group was 59.23 HU/mm as compared to 82.32 HU/mm in the failure group (p 0.007). There was 88.1% success rate in HU density < 76 HU/mm as compared to 65.6% in HU density > 76 HU/mm (p 0.020). After multivariate analysis, stone density failed to show any statistical significance. Hence, it does not appear to be a good tool to use if simple Hounsfield unit value of stone is considered.

## Conclusions

ESWL is an effective noninvasive treatment modality for ureteric stones in carefully selected patients. NCCT not only helps in diagnosing the urinary stones, but it also assists in the prediction of ESWL success. BMI, SSD, stone Hounsfield units, Hounsfield unit density and stone area are simple to calculate and can be used to predict the outcome of ESWL for ureteric stones. Patients having ureteric stones with BMI > 30 kg/m2, Hounsfield units > 1000 HU, HD > 76 HU/mm, larger stone area, and longer SSD should be discouraged for ESWL treatment.
